# Molecular Mechanisms and Therapeutic Potential of Baicalein in Acute Pancreatitis: A Comprehensive Review

**DOI:** 10.3390/biom16010151

**Published:** 2026-01-14

**Authors:** Linbo Yao, Shiyu Liu, Wei Huang, Xinmin Yang

**Affiliations:** 1 West China Centre of Excellence for Pancreatitis, Institute of Integrated Traditional Chinese and Western Medicine, West China-Liverpool Biomedical Research Centre, West China Hospital, Sichuan University, Chengdu 610041, China; linbo_yao@stu.scu.edu.cn (L.Y.); shiyuliu@wchscu.cn (S.L.); 2 West China Biobank, West China Hospital, Sichuan University, Chengdu 610041, China

**Keywords:** acute pancreatitis, baicalein, inflammatory cascades, immune microenvironment, pyroptosis, ferroptosis, apoptosis, extracellular histones, pancreatic lipase, clinical translation

## Abstract

Acute pancreatitis (AP) is a severe inflammatory disorder characterized by a complex molecular pathophysiology involving premature zymogen activation, organelle dysfunction, and systemic immune dysregulation. Current therapeutic strategies remain largely supportive, underscoring the critical need for specific molecular-targeted interventions. Baicalein, a bioactive flavonoid derived from *Scutellaria baicalensis* Georgi, has emerged as a potent pleiotropic agent. This review comprehensively synthesizes the molecular mechanisms underlying baicalein’s therapeutic efficacy in AP. Its capacity to intercept the pathological cascade at multiple checkpoints is elucidated, from mitigating the initiating cytosolic calcium overload and preserving mitochondrial integrity to suppressing the cytokine storm via the TLR4/NF-κB/MAPK signaling axis. Crucially, baicalein modulates the pancreatic immune microenvironment by driving the phenotypic polarization of macrophages from pro-inflammatory M1 to reparative M2 states and regulating neutrophil dynamics, specifically by inhibiting infiltration and neutrophil extracellular trap formation. Furthermore, its role in orchestrating regulated cell death pathways is highlighted, specifically by blocking pyroptosis and ferroptosis while modulating apoptosis, and its function as a biophysical scavenger of circulating histones and pancreatic lipase to neutralize systemic toxins. Consequently, this review emphasizes the multi-target biological activities of baicalein, providing a mechanistic rationale for its development as a precision therapeutic candidate for AP.

## 1. Introduction

### 1.1. Pathophysiology of Acute Pancreatitis

Acute pancreatitis (AP) represents a prevalent gastrointestinal emergency manifesting as a clinical spectrum that extends from mild, self-limiting episodes to severe, life-threatening conditions, with its global incidence exhibiting a concerning upward trend [1,2]. The pathophysiology of AP involves a complex and dynamic cascade that initiates with localized tissue injury and progresses toward systemic catastrophe [3,4]. Precipitated by diverse insults such as gallstones, alcohol abuse, or hypertriglyceridemia, the fundamental pathogenic event involves the premature intracellular activation of digestive zymogens, particularly trypsinogen, within pancreatic acinar cells (PACs) [3]. This intra-acinar activation ignites a cycle of pancreatic autodigestion, acinar cell injury, and eventual cell death [5,6]. Crucially, this autodigestive machinery is driven by intracellular calcium (Ca^2+^) overload in PACs, a pivotal initiating mechanism that subsequently precipitates mitochondrial dysfunction, endoplasmic reticulum (ER) stress, and impaired autophagic flux [7]. These Ca^2+^-dependent processes serve as the central axis connecting local cellular injury to the broader systemic inflammation and multiple organ injury of AP [8].

Following cellular necrosis, damaged acinar cells release their contents, including digestive enzymes and a host of endogenous signaling molecules known as damage-associated molecular patterns (DAMPs) and alarmins [9]. Upon release, DAMPs such as high mobility group box 1 (HMGB1) and extracellular histones, activate resident immune cells, primarily pancreatic macrophages, and trigger the recruitment of circulating leukocytes like neutrophils to the injury site [10,11,12]. This immune activation instigates an intense inflammatory response, often termed a “cytokine storm”, which is characterized by the massive production and release of pro-inflammatory cytokines, including tumor necrosis factor-alpha (TNF-α), interleukin-6 (IL-6), and interleukin-1 beta (IL-1β), along with various chemokines [13,14,15]. This inflammatory response exacerbates vascular permeability, leading to pancreatic edema, ischemia, and further parenchymal necrosis [16]. In approximately 20% of cases, the localized pancreatic inflammation spills over into the systemic circulation, triggering a systemic inflammatory response syndrome (SIRS) [17]. SIRS acts as the primary driver of severe AP and mortality [18,19]. The systemic dissemination of cytokines and DAMPs induces widespread endothelial dysfunction, vascular leakage, and hypovolemia, culminating in multiple organ dysfunction syndrome (MODS) [20]. Particularly, the lungs are typically the first and most common organ to fail, leading to acute lung injury (ALI) or its more severe form, acute respiratory distress syndrome (ARDS), which remains a leading cause of death in AP patients [21].

### 1.2. Current Therapeutic Limitations

While current understanding of AP pathophysiology has deepened significantly, clinical management of AP remains profoundly challenging. Treatment strategies are predominantly supportive and non-specific, focusing on managing complications rather than halting the underlying disease process [22]. The standard care relies on aggressive intravenous fluid resuscitation to sustain organ perfusion, bowel rest followed by early nutritional support, and comprehensive pain control [23]. In severe cases, therapeutic management is escalated to intensive care monitoring for organ failure and, if pancreatic necrosis becomes infected, antibiotic therapy or invasive (endoscopic or surgical) debridement [22]. This supportive-care paradigm underscores a critical “therapeutic gap” defined by the complete absence of pharmacological agents approved to interrupt the early, critical events of AP [24]. The multifaceted nature of AP, which intertwines enzymatic activation, inflammation, oxidative stress, and programmed cell death, creates a narrow and difficult temporal window for intervention. Consequently, a “magic bullet” targeting a single pathway is unlikely to succeed; effective therapy demands pleiotropic agents capable of simultaneously modulating multiple pathological pathways. This urgent clinical unmet need has driven the investigation into multi-target natural compounds.

### 1.3. Therapeutic Potential of Baicalein

**Figure 1:**
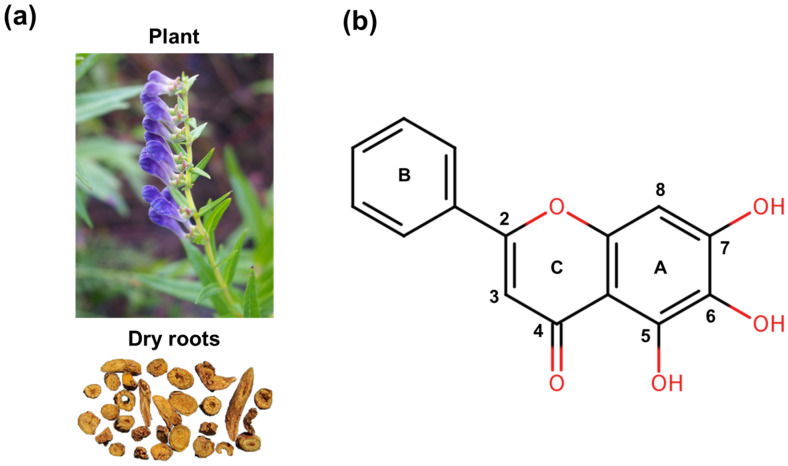
Structures of baicalein derived from *Scutellaria baicalensis* Georgi: (**a**) *Scutellaria baicalensis* plant and its dry roots. (**b**) Chemical structure of baicalein.

Baicalein is a prominent bioactive flavonoid primarily found in the roots of *Scutellaria baicalensis* Georgi (Figure 1). This herbal source, known as “Huangqin” in traditional Chinese medicine (TCM), has been utilized for centuries to treat inflammatory diseases, infections, and hypertension [25]. Notably, it is widely distributed in the genus *Scutellaria* and various edible plants, such as *Oroxylum indicum* and *Thymus vulgaris* (Thyme), suggesting a favorable natural safety profile for clinical application [26,27]. Modern pharmacological studies have validated these traditional applications, demonstrating that baicalein possesses a remarkable breadth of biological effects, including potent anti-inflammatory, antioxidant, anti-pyroptotic, antimicrobial, lipid metabolism-regulating and anti-tumor properties [26,28]. Moreover, the compound exhibits significant multi-organ protection, including neuroprotective, cardioprotective, respiratory protective, gastroprotective, hepatoprotective, and renoprotective effects [26]. While various flavonoids such as quercetin, genistein, apigenin, kaempferol, naringenin, and hesperidin have demonstrated protective effects in experimental AP, primarily through shared anti-inflammatory and antioxidant mechanisms, baicalein appears to offer distinct therapeutic advantages [29]. Beyond these common properties, preclinical studies indicate that baicalein regulates cell death modes and inhibits human pancreatic lipase activity [30]. This broader efficacy profile allows for a more comprehensive mitigation of disease severity compared to related compounds, positioning baicalein as a particularly promising candidate for AP management.

Crucially, the pharmacological action of baicalein *in vivo* is defined by a dynamic “bidirectional metabolic shuttle” with its glycoside, baicalin [31]. Following oral administration and absorption, baicalein undergoes extensive first-pass metabolism, where it is rapidly reconjugated by hepatic UDP-glucuronyltransferases into baicalin and other metabolites [31]. These conjugates are subsequently excreted via multidrug resistance-associated protein 2 into the bile. Within the intestine, gut microbiota-derived β-glucuronidase hydrolyzes the biliary baicalin back into the absorbable aglycone [31]. This enterohepatic circulation serves as a circulating reservoir, continuously regenerating the bioactive baicalein and thereby extending its systemic half-life and therapeutic duration. Growing preclinical evidence indicates that baicalein can effectively target the key pathological processes of AP at multiple levels. This review aims to comprehensively synthesize the current scientific evidence regarding the multifaceted mechanisms of baicalein in AP, critically evaluate its therapeutic potential, and discuss the pharmacological challenges that must be overcome for its clinical translation.

## 2. Suppression of Inflammatory Cascades

Inflammation serves as the pivotal engine of AP pathogenesis, driving the pathological progression from localized pancreatic injury to systemic organ failure [32]. The most extensively documented therapeutic effect of baicalein is its potent suppression of this inflammatory cascade via a multi-layered mechanism.

### 2.1. Blockade of the TLR4/NF-κB/MAPK Signaling Axis

Baicalein’s ability to suppress the cytokine storm stems not from a single-target interaction, but from its capacity to orchestrate a comprehensive blockade of the intracellular signaling axis, intercepting inflammatory transmission from the cell surface receptors down to the nuclear transcription factors. At the upstream sensor level, baicalein targets Toll-like receptor 4 (TLR4), the critical pattern recognition receptor activated by both exogenous pathogen-associated molecular patterns (PAMPs) (like lipopolysaccharide from a compromised gut barrier) and endogenous DAMPs released from necrotic cells [33]. TLR4 activation is a major trigger for the inflammatory cascade via two main adaptors, myeloid differentiation primary response gene 88 (MyD88) and TIR-domain-containing adapter-inducing interferon-β (TRIF). Baicalein exerts significant therapeutic effects by interfering with this crucial initiating step. Mechanistically, it upregulates microRNA-182 (miR-182), which directly targets TLR4 mRNA to suppress its expression [34]. Experimental data confirm that miR-182 mimics potentiate baicalein’s anti-inflammatory effects, whereas inhibitors partially reverse them, establishing the miR-182/TLR4 axis as a central target. This inhibition translates into tangible pathophysiological improvements; in AP-associated ALI models, baicalein markedly suppresses the expression of TLR4, MyD88, and TRIF, directly correlating with attenuated pulmonary edema and inflammatory infiltration [35].

**Figure 2:**
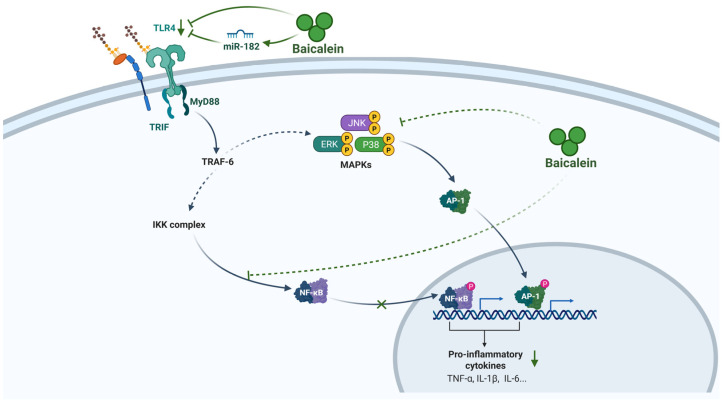
Anti-inflammatory mechanism of baicalein in AP. Baicalein suppresses the inflammatory response in AP by orchestrating a multi-target blockade of the TLR4/NF-κB/MAPK signaling axis and attenuating the production of key pro-inflammatory cytokines. It upregulates miR-182 to inhibit TLR4 expression and downstream MyD88/TRIF adaptors, concurrently blocks NF-κB nuclear translocation by stabilizing IκB, and inhibits MAPK activation. This comprehensive transcriptional blockade leads to a profound reduction in cytokines (e.g., TNF-α, IL-1β, IL-6). Abbreviations: TLR4, Toll-like receptor 4; miR, microRNA; MyD88, myeloid differentiation primary response 88; TRIF, TIR-domain-containing adapter-inducing interferon-β; TRAF-6, TNF receptor-associated factor 6; IKK, IκB kinase; NF-κB, nuclear factor kappa B; MAPK, mitogen-activated protein kinase; ERK, extracellular signal-regulated kinase; JNK, c-Jun N-terminal kinase; AP-1, Activator protein 1; TNF-α, tumor necrosis factor-alpha; IL-1β, interleukin-1 beta; IL-6, interleukin-6.

Following the blockade of upstream sensors, baicalein further arrests the propagation of signals to downstream transcriptional effectors. The primary target is arguably the nuclear transcription factor-kappa B (NF-κB) pathway, the “master switch” controlling the transcription of numerous pro-inflammatory cytokines, chemokines, and adhesion molecules. In resting cells, NF-κB dimers are sequestered in the cytoplasm by inhibitory IκB proteins [36]. Baicalein effectively inhibits the phosphorylation and proteasomal degradation of IκB, thereby preventing the liberation and nuclear translocation of the NF-κB p65 subunit [37]. By locking NF-κB in the cytoplasm, baicalein effectively abrogates the transcription of a broad spectrum of cytokine genes [38]. Critically, this action is bolstered by the concurrent inhibition of synergistic pathways, including the mitogen-activated protein kinase (MAPK) cascade (p38, extracellular signal-regulated kinase (ERK), and c-Jun N-terminal kinases (JNK)) and the Janus Kinase 2 (JAK2)/signal transducer and activator of transcription 3 (STAT3) axis [36,38]. This multi-pronged intervention, spanning from the miR-182-mediated suppression of TLR4 sensing to the direct inhibition of NF-κB and MAPK activation, creates a robust transcriptional blockade. This mechanism prevents the amplification of inflammatory signaling, thereby accounting for the profound reduction in systemic cytokine levels observed in severe AP (Figure 2).

### 2.2. Attenuation of Pro-Inflammatory Cytokines

A hallmark of AP is the rapid and excessive production of pro-inflammatory cytokines, which amplify tissue damage and mediate systemic toxicity. Baicalein has been shown to be a robust inhibitor of this cytokine storm in a variety of preclinical models. Its anti-inflammatory activity was confirmed *in vitro* in lipopolysaccharide-induced AR42J cells (a rat PACs line), where baicalein treatment markedly decreased cytokine levels [37]. This therapeutic efficacy was further validated *in vivo* in both a sodium taurocholate-induced severe AP rat model and a repeated cerulein injection-induced AP mouse model [38,39]. Mechanistically, baicalein-treated animals exhibited a significant decrease in the expression levels of the key inflammatory mediators, including TNF-α, IL-1β and IL-6 in the pancreatic tissue. The downregulation of these pro-inflammatory cytokines by baicalein not only alleviates local pancreatic injury but also plays a crucial role in limiting the development of SIRS. *In vivo* models of severe AP-ALI have shown that baicalein administration decreases cytokine levels in both pancreatic and lung tissues as well as bronchoalveolar lavage fluid (BALF) [35]. This dual protective effect is further corroborated by a marked reduction in serum cytokine concentrations, demonstrating that the anti-inflammatory properties of baicalein translate to substantial systemic benefits [40,41].

## 3. Modulation of the Immune Microenvironment

In AP, neutrophils are rapidly recruited and act as primary instigators of tissue damage and inflammation through the release of reactive oxygen species, proteases, and cytokines [42]. Concurrently, monocytes/macrophages are recognized as the key contributors to initiating and driving systemic inflammation, forming the main inflammatory cell population in the pancreas [43]. Therefore, targeting the pathogenic functions of these innate immune cells is a critical therapeutic strategy for mitigating disease severity. Baicalein imposes a comprehensive check on the cellular effectors and redirects the pancreatic immune microenvironment from a pro-inflammatory state toward a reparative phenotype in AP [35], primarily by regulating macrophage plasticity and restricting neutrophil invasion [36,37].

### 3.1. Fine-Tuning of Macrophages

**Figure 3:**
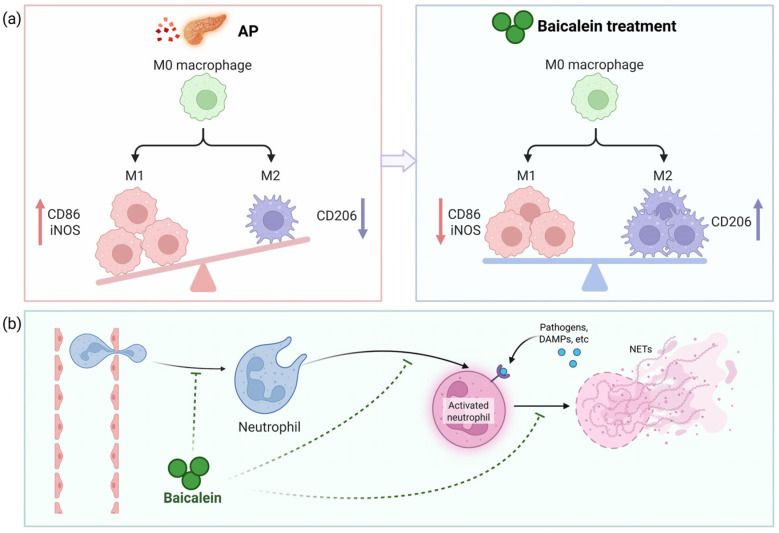
Immunomodulatory mechanism of baicalein in AP. Baicalein ameliorates AP by reprogramming the pancreatic immune microenvironment: (**a**) It fine-tunes macrophage polarization from a pro-inflammatory M1 phenotype to a pro-reparative M2 state; (**b**) Baicalein limits neutrophil-mediated damage by suppressing infiltration, inhibiting activation, and impeding neutrophil extracellular trap (NET) formation. This coordinated regulation of innate immune cells shifts the balance from tissue destruction to resolution. Abbreviations: M1, classically activated macrophage; iNOS, inducible nitric oxide synthase; M2, alternatively activated macrophage; CD, cluster of differentiation; DAMPs, damage-associated molecular patterns; NETs, neutrophil extracellular traps.

Baicalein induces the phenotypic switch of macrophages between the pro-inflammatory M1 and pro-reparative M2 states, a process that governs the balance between inflammation and tissue repair following acute pancreatitis [12,44]. By inhibiting the TLR4/NF-κB pathway, which is essential for M1 commitment, baicalein represses the expression of M1 surface markers (e.g., CD86 and inducible nitric oxide synthase (iNOS)) while concomitantly promoting the expression of M2 markers (e.g., CD206) [45,46]. Additionally, baicalein achieves macrophage polarization by specifically downregulating the arachidonate 15-lipoxygenase (ALOX15) pathway, which is intrinsically linked to the regulation of ferroptosis [47]. This ‘M1-to-M2’ shift induced by baicalein is critical, as it transforms the local immune milieu from one that perpetuates tissue destruction to one that facilitates debris clearance and tissue resolution (Figure 3a).

### 3.2. Modulation of Neutrophils

Baicalein constructs a defensive barrier against neutrophil-mediated destruction. Neutrophils are the first responders to migrate into the pancreas, where they exacerbate injury through the release of myeloperoxidase (MPO) and the formation of neutrophil extracellular traps (NETs) [10,48,49]. Elevated MPO activity is a hallmark of neutrophil recruitment and correlates with tissue damage severity [50]. Baicalein exerts a dual inhibitory effect on this process. At the vascular interface, it downregulates the expression of adhesion molecules (such as intercellular adhesion molecule-1 (ICAM-1)), thereby reducing the physical sequestration of neutrophils in both pancreatic and pulmonary tissues [51]. Furthermore, baicalein impedes the formation of NETs [52]. By limiting both the recruitment of neutrophils and their extracellular trapping capacity, baicalein effectively blunts the ‘second hit’ of the inflammatory cascade (Figure 3b).

## 4. Inhibition of Regulated Cell Death

While inflammation propels the progression of AP, the continuous depletion of PACs provides the necessary substrate for disease perpetuation. Contemporary research underscores that this parenchymal destruction is not merely necrotic but is driven by a sophisticated interplay of programmed cell death pathways, including pyroptosis, ferroptosis, and apoptosis. These pathways do not operate in isolation but collectively exacerbate the inflammatory milieu, creating a cycle of tissue injury.

### 4.1. Attenuation of Cytosolic Calcium Overload

**Figure 4:**
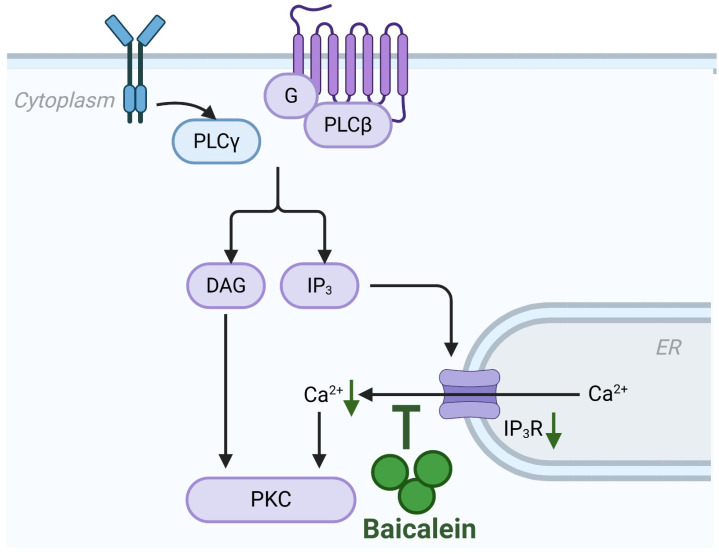
Inhibition of calcium overload by baicalein in AP. Baicalein attenuates the pathological cytosolic calcium overload that initiates acinar cell injury. It downregulates the expression of IP_3_R on the endoplasmic reticulum, thereby inhibiting excessive calcium release. Abbreviations: ER, endoplasmic reticulum; IP_3_R, inositol 1,4,5-trisphosphate receptor.

Although premature zymogen activation is the enzymatic hallmark of AP, the pathological cascade is fundamentally ignited by aberrant, sustained elevations in cytosolic calcium [7]. This intracellular calcium overload acts as the primary trigger for acinar cell injury, precipitating a triad of cellular crises: mitochondrial dysfunction, ER stress, and impaired autophagic flux [8]. While physiological calcium oscillations regulate enzyme secretion, pathological stimuli (e.g., cholecystokinin, bile acids or fatty acids (FAs)) induce a toxic calcium overload that drives the depolarization of mitochondria, the premature, intra-acinar activation of digestive zymogens and activation of cell death pathway [8]. Baicalein exerts a profound “upstream” protective effect by stabilizing this calcium dynamics at the precise moment of initiation. Mechanistically, baicalein has been shown to directly counteract the sodium taurocholate-induced surge in cytosolic calcium by downregulating the expression of the inositol 1,4,5-trisphosphate receptor (IP_3_R), the principal calcium-release channel on the ER [40]. By restricting IP_3_R-mediated calcium release, baicalein prevents the formation of large cytoplasmic vacuoles, a morphological hallmark of acinar injury, and blocks the pathological colocalization of zymogen granules with lysosomes [40]. Consequently, this intervention halts the autodigestive process before it can propagate necrosis or downstream cell death pathways that trigger an inflammatory cytokine storm (Figure 4).

### 4.2. Inhibition of Pyroptosis

Pyroptosis is a lytic form of programmed cell death executed by gasdermins, which plays crucial roles in inflammation [53]. The lysis of PACs releases DAMPs that trigger inflammasome activation in adjacent cells, which in turn amplifies IL-1β/IL-18 release, propagates tissue necrosis, and ultimately sustains a vicious feed-forward loop in AP [54]. Baicalein dismantles this cycle through a dual-layered intervention targeting both protein interactions and transcriptional networks. Molecular docking and drug affinity responsive target stability (DARTS) assays have confirmed that baicalein directly binds to the alarmin HMGB1 [46]. This physical interaction is critical, as it effectively disrupts the pathological HMGB1/TLR4/NOD-like receptor family pyrin domain containing 3 (NLRP3) signaling axis [55]. This action abrogates inflammasome priming and concurrently suppresses M1 macrophage polarization, as indicated by decreased CD86 and iNOS expression [46]. Consequently, the disruption of this axis leads to reduced expression of key inflammasome components, including NLRP3, ASC, cleaved caspase-1, and the pore-forming GSDMD-N fragment [46]. This mechanism is further validated by the observation that HMGB1 overexpression partially negates baicalein’s protective effects [46].

In addition to inhibiting HMGB1, baicalein can modulate specific microRNA networks to reinforce the suppression of pyroptosis. In cellular models of AP, baicalein significantly upregulates miR-224-5p, which functions as a molecular brake by simultaneously targeting distinct pathological axes. It suppresses the NLRP3/ASC/caspase-1/GSDMD inflammasome axis to limit pyroptotic execution, while concomitantly inhibiting the poly (ADP-ribose) polymerase 1 (PARP1)/NF-κB signaling cascade [37]. The latter effect is particularly important because reduced PARP1 activity diminishes NF-κB p65 nuclear translocation, thereby lowering the transcriptional priming of pro-inflammatory genes such as NLRP3, a mechanism that further dampens the pyroptotic cycle. Further broadening its regulatory scope, baicalein targets the thioredoxin-interacting protein (TXNIP), a critical binding partner required for NLRP3 inflammasome assembly. By upregulating miR-192-5p, baicalein promotes the degradation of TXNIP mRNA, effectively severing the physical interaction between TXNIP and NLRP3 necessary for oligomerization. This protective mechanism has been specifically validated in models of hyperlipidemic AP [56].

**Figure 5:**
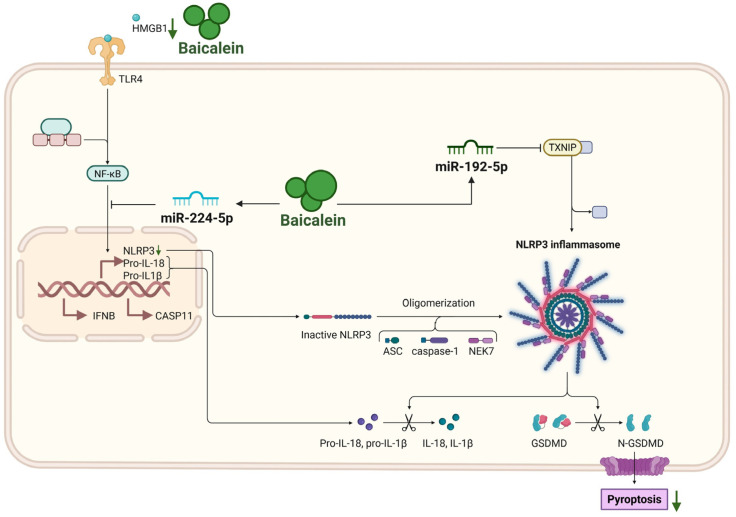
Inhibition of pyroptosis by baicalein in AP. Baicalein inhibits pyroptosis of pancreatic acinar cells through a dual strategy. It directly binds to HMGB1 to disrupt the HMGB1/TLR4/NLRP3 inflammasome axis, and upregulates miR-224-5p and miR-192-5p to suppress the NF-κB and TXNIP/NLRP3 pathways, respectively. This coordinated inhibition reduces the expression of NLRP3, ASC, cleaved caspase-1, and GSDMD-N, thereby attenuating pyroptotic cell death and the release of associated cytokines (IL-18, IL-1β). Abbreviations: HMGB1, high mobility group box 1; NLRP3, NOD-like receptor family pyrin domain containing 3; IFNB, Interferon-beta; CASP11, caspase-11; ASC, apoptosis-associated speck-like protein containing a CARD; TXNIP, thioredoxin-interacting protein; NEK7, NIMA-related kinase 7; GSDMD, gasdermin D; N-GSDMD, N-terminal fragment of GSDMD.

Collectively, the dual-pronged mechanism, blocking extracellular triggers via the HMGB1 axis and dismantling intracellular activators (NF-κB and TXNIP) via miRNA modulation, converges to potently attenuate pyroptotic cell death. Functionally, this manifests as enhanced cell viability and a profound reduction in the release of pyroptosis-associated cytokines, including IL-1β, IL-6, IL-18, and TNF-α [37,46], highlighting baicalein’s multifaceted role in mitigating inflammatory cell death (Figure 5).

### 4.3. Mitigation of Ferroptosis

**Figure 6:**
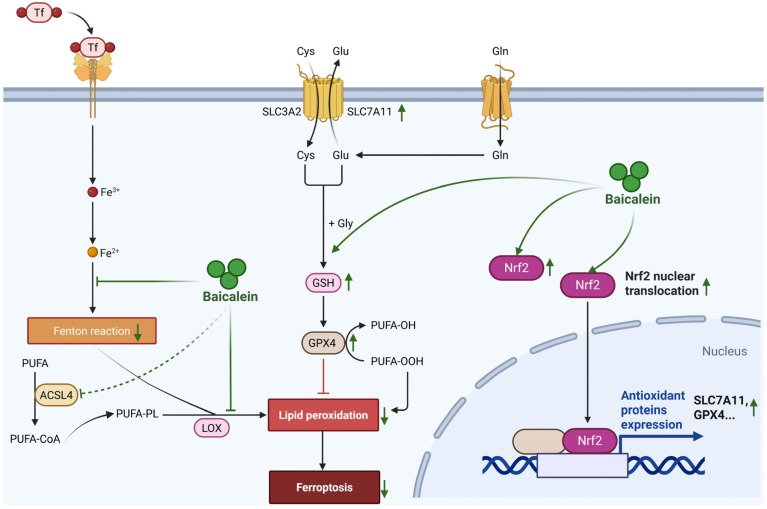
Mitigation of ferroptosis by baicalein in AP. Baicalein mitigates ferroptosis through a multi-targeted defense strategy. It activates the Nrf2 antioxidant pathway to upregulate SLC7A11 and GPX4, thereby restoring the cellular glutathione-dependent defense system and detoxifying lipid peroxides. Concurrently, baicalein acts as an iron chelator to sequester the labile iron pool, thus inhibiting the iron-catalyzed Fenton reaction. Furthermore, it may suppress the lipid-modifying enzyme ACSL4, limiting the availability of peroxidation-prone substrates. This multi-layered intervention collectively establishes a comprehensive blockade against ferroptosis of pancreatic acinar cells. Abbreviations: Tf, Transferrin; PUFA, polyunsaturated fatty acid; PUFA-CoA, polyunsaturated fatty acyl-CoA; PUFA-OH, hydroxylated polyunsaturated fatty acid; PUFA-OOH, hydroperoxylated polyunsaturated fatty acid; ACSL4, long-chain-fatty-acid-CoA ligase 4; LOX, lipoxygenase; SLC3A2, solute carrier family 3 member 2; SLC7A11, solute carrier family 7 member 11; Cys, cysteine; Glu, glutamate; Gln, glutamine; Gly, glycine; GSH, glutathione; GPX4, glutathione peroxidase 4; Nrf2, nuclear factor erythroid 2-related factor 2.

In parallel to pyroptosis, baicalein critically mitigates ferroptosis, a process inextricably linked to oxidative stress in AP [57]. Distinct from the inflammatory lytic nature of pyroptosis, ferroptosis represents an iron-dependent form of regulated cell death driven by the catastrophic accumulation of lipid peroxides [58]. Although direct investigations into baicalein’s anti-ferroptosis in AP are evolving, its established pharmacological profile suggests a potent intervention at the core of this lethal machinery. Notably, baicalein has been widely validated as a potent ferroptosis inhibitor in various hyper-inflammatory and ischemic conditions, including specific evidence demonstrating its ability to block erastin-induced ferroptosis of PACs lines [59]. Mechanistically, baicalein functions as a dual-targeting agent against the core machinery of ferroptosis. First, it activates the nuclear factor erythroid 2-related factor 2 (Nrf2) signaling pathway, which serves as a key regulator of cellular antioxidant defense [60]. Upon activation, Nrf2 translocates to the nucleus to drive the transcription of solute carrier family 7 member 11 (SLC7A11) and glutathione peroxidase 4 (GPX4). Baicalein effectively restores Nrf2/SLC7A11/GPX4 axis, thereby replenishing the cellular ‘antioxidant buffer’ and converting toxic lipid hydroperoxides into benign alcohols [61,62]. Second, baicalein directly regulates iron metabolism. Its flavonoid structure enables it to act as a natural iron chelator, sequestering the labile iron pool within PACs [63]. This chelation effectively blocks the Fenton reaction, preventing the conversion of hydrogen peroxide into highly reactive hydroxyl radicals [64]. Additionally, recent insights suggest that baicalein may downregulate long-chain-fatty-acid-CoA ligase 4 (ACSL4), the enzyme responsible for enriching membranes with peroxidation-prone polyunsaturated fatty acids (PUFAs) [65]. By simultaneously reinforcing enzymatic defenses, sequestering catalytic iron, and limiting lipid substrate availability, baicalein imposes a multi-layered blockade against ferroptosis (Figure 6).

### 4.4. Modulation of Apoptosis

Apoptosis in AP represents a silent but critical driver of parenchymal loss, primarily driven by mitochondrial and ER dysfunction. Baicalein exerts a robust cytoprotective effect by intercepting these organelle-specific lethal signals. First, baicalein targets the mitochondrial execution pathway, which is centrally implicated in AP. In PACs, intrinsic apoptosis is ignited by mitochondrial outer membrane permeabilization (MOMP), a catastrophic event that leads to the release of mitochondrial proteins such as cytochrome C and diablo IAP-binding mitochondrial protein (DIABLO, also known as Smac), which subsequently activates the initiator caspase-9 [57]. Baicalein treatment effectively arrests this cascade at the mitochondrial checkpoint. Mechanistically, baicalein recalibrates the Bcl-2 family rheostat by upregulating anti-apoptotic Bcl-2 and repressing pro-apoptotic Bax, thereby preventing the MOMP-dependent release of cytochrome C described in AP pathology [66]. By stabilizing mitochondrial integrity, baicalein effectively aborts the assembly of the apoptotic body and silences the downstream executioner caspases [67].

**Figure 7:**
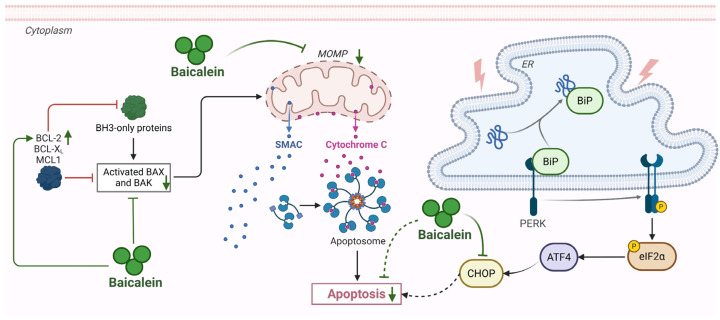
Modulation of apoptosis by baicalein in AP. Baicalein exerts cytoprotective effects in AP by intercepting key apoptotic pathways. It upregulates anti-apoptotic BCL-2 and downregulates pro-apoptotic BAX to stabilize MOMP, preventing cytochrome C release and caspase-9 activation. Concurrently, baicalein suppresses ER stress-induced apoptosis by inhibiting the CHOP/caspase-12 pathway. Abbreviations: BCL-2, B-cell lymphoma 2; BCL-X_L_, B-cell lymphoma-extra large; MCL1, myeloid cell leukemia 1; BAX, Bcl-2-associated X protein; BAK, BCL-2 antagonist/killer; MOMP, mitochondrial outer membrane permeabilization; BiP, binding immunoglobulin protein; PERK, protein kinase R-like endoplasmic reticulum kinase; eIF-2α, eukaryotic initiation factor 2 alpha; CHOP, C/EBP homologous protein.

Second, baicalein addresses the ER stress-induced apoptosis to protect against pancreatic injury. Due to the high rate of protein synthesis in acinar cells, the pancreas is particularly susceptible to ER stress during AP, where the accumulation of unfolded proteins (e.g., trypsinogen) exceeds the organelle’s folding capacity [3]. When this stress persists, cellular mechanisms shift from adaptive protection to induced apoptosis to eliminate damaged cells [3,68]. Baicalein counteracts this pathological process by restoring proteostatic balance. Baicalein suppresses the detrimental C/EBP homologous protein (CHOP)/caspase-12 pathway within the unfolded protein response [69,70]. By blocking this specific signal, it prevents over-stressed acinar cells from being pushed into programmed cell death. This aligns with evidence that phytoceuticals can alleviate AP specifically by regulating acinar cell apoptosis and restoring intracellular homeostasis [57,71]. However, the role of apoptosis in AP remains complex and context-dependent. While often considered less inflammatory than necrosis, excessive apoptosis can lead to the critical loss of functional acinar mass, and uncleared apoptotic bodies may progress to secondary necrosis, subsequently releasing DAMPs such as histones and HMGB1 to propagate inflammation [71]. Consequently, whether the induction or inhibition of apoptosis is definitively beneficial in the clinical environment remains to be confirmed. In this context, baicalein’s mechanism offers a distinct advantage: by preventing the upstream commitment to cell death, it preserves functional acinar mass while simultaneously limiting the potential secondary release of apoptosis derived DAMPs that could otherwise exacerbate the inflammatory milieu (Figure 7).

### 4.5. Modulation of Emerging Non-Canonical Cell Death Pathways

The evolving landscape of cell death research in AP has extended beyond classical modalities to encompass novel, non-canonical pathways. Among these, parthanatos and cuproptosis represent emerging frontiers that, while requiring further validation, offer distinct therapeutic targets for pleiotropic agents like baicalein.

Parthanatos constitutes a distinct modality of regulated necrosis, fundamentally characterized by the hyperactivation of PARP1 in response to severe genomic stress [72]. This pathological overactivation precipitates a “bioenergetic catastrophe” through the rapid exhaustion of cytosolic NAD^+^ and ATP, culminating in mitochondrial depolarization and the nuclear translocation of apoptosis-inducing factor (AIF) [73]. While Nagy-Pénzes et al. have postulated that PARP1 exacerbates AP pathology by amplifying oxidative stress, definitive experimental validation explicitly linking this pathway to pancreatic acinar cell loss remains to be fully solidified [74]. Nevertheless, the mechanistic convergence of oxidative damage and metabolic collapse in AP offers a compelling rationale for baicalein intervention. By inhibiting the activity of PARP1, baicalein is strategically positioned to prevent the energy depletion and AIF-mediated execution inherent to this cascade, providing a cytoprotective mechanism that complements its anti-inflammatory efficacy [75].

Parallel to this, cuproptosis has recently been defined as a form of mitochondrial cell death triggered by the aberrant accumulation of intracellular copper, which leads to the toxic aggregation of lipoylated TCA cycle enzymes [76]. Preliminary investigations in models of L-arginine-induced AP have indicated that limiting copper bioavailability can attenuate pancreatic injury, hinting at a pathogenic role for copper dyshomeostasis [77,78]. While the precise contribution of cuproptosis to AP progression remains to be fully elucidated, the chemical structure of baicalein provides a unique advantage. As a flavonoid with inherent metal-chelating properties, baicalein may mitigate copper-induced proteotoxicity, offering a potential protective strategy against this metal-dependent cell death.

## 5. Neutralization of Circulating Toxins

The transition from local pancreatic injury to MODS is driven by the systemic dissemination of toxic mediators. Following the death of acinar cells (as detailed in Part 4), a barrage of intracellular contents, including enzymatic toxins (lipase) and nuclear proteins (histones), leak into the circulation. These circulating toxins act as the fuel for systemic inflammation and organ failure. Baicalein exerts a unique therapeutic effect at this stage, acting not only as a cellular signaling modulator but also as a direct scavenger of these systemic poisons, thereby shielding distal organs from chemical and biophysical injury.

### 5.1. Neutralization of Circulating Histones

During AP, the breakdown of the gut barrier facilitates the leakage of toxic mediators into the mesenteric lymph. Among these, extracellular histones, which originate not only from necrotic acinar cells but also from apoptotic lymphocytes in the gut lymph, have emerged as critical DAMPs [79]. Upon entering the systemic circulation, these cationic nuclear toxins bind avidly to the negatively charged plasma membranes of vascular endothelial cells, particularly pulmonary microvascular endothelial cells [80]. This electrostatic interaction triggers cytotoxicity through direct membrane disruption and aberrant intracellular calcium influx, subsequently precipitating thrombosis and distant organ injury [9]. A breakthrough mechanism reveals that baicalein functions as a high-affinity biophysical scavenger of these toxic proteins [41]. Supported by biolayer interferometry and molecular dynamics simulations, baicalein forms stable hydrogen bonds with key amino acid residues on the histone surface, thereby abrogating histone accumulation on endothelial membranes and blocking the subsequent calcium overload and oxidative stress [41]. By inhibiting the toxicity of histones in the bloodstream and lymph, baicalein significantly attenuates endothelial necrosis, providing a direct explanation for its efficacy in mitigating cardiac, hepatic, and renal injury in severe AP models [41]. This mechanism represents a distinct protective layer beyond its classical intracellular anti-inflammatory signaling.

### 5.2. Inhibition of Pancreatic Lipase

In addition to nuclear toxins, the leakage of activated pancreatic lipase is a hallmark of AP pathophysiology, particularly in the context of hyperlipidemic AP [81,82]. The aberrant activity of lipase in the interstitium and systemic circulation hydrolyzes triglycerides into excessive free fatty acids (FFAs) [81,83,84]. These FFAs, particularly free unsaturated fatty acids, exert potent lipotoxicity, inducing mitochondrial damage, calcium overload, and further cell necrosis in peri-pancreatic adipose tissue (saponification) and distant organs [85,86,87]. Calcium overload synergizes with FFA-induced mitochondrial depolarization, thereby triggering accelerated acinar cell death and subsequent systemic organ injury [87]. Baicalein has been shown to act as a specific and potent inhibitor of human pancreatic lipase activity (IC_50_ = 2.19 μM) [30,88]. It binds to an allosteric pocket via its A-ring pyrogallol moiety, hindering the enzyme’s activation and thereby reducing the pathological generation of cytotoxic FFAs. This blockade interrupts the vicious cycle of “lipotoxicity-inflammation,” wherein FFAs would otherwise serve as potent pro-inflammatory ligands (via TLR4) to drive M1 macrophage polarization [45,89]. Consequently, this inhibition alleviates the burden of fat necrosis, thereby reducing the metabolic toxicity that drives visceral organ failure [83].

### 5.3. Reactive Oxygen Species Scavenging and Redox Homeostasis

Oxidative stress, characterized by an imbalance between the production of reactive oxygen species (ROS) and the biological system’s ability to detoxify reactive intermediates, plays a critical role in the pathogenesis of AP [90,91]. Baicalein exerts a potent, dual-layered antioxidant effect to counteract this pathological state [92]. Firstly, it functions as a direct free radical scavenger. Structural activity relationship studies indicate that the specific arrangement of three hydroxyl groups on the A-ring (C5, C6, and C7) of baicalein confers exceptional electron-donating capabilities, enabling the direct neutralization of superoxide anions, hydroxyl radicals, and hydrogen peroxide [93]. While this enables the direct neutralization of radicals *in vitro*, recent redox biology consensus suggests that such direct scavenging is kinetically limited *in vivo* due to low bioavailability compared to endogenous antioxidants like glutathione [94]. Secondly and more significantly, baicalein reinforces the cellular defense machinery by upregulating endogenous antioxidants. This is considered the primary mechanism underlying its therapeutic efficacy. It acts as a pharmacological activator of Nrf2 signaling pathway. By dissociating Nrf2 from its inhibitor Keap1 and facilitating its nuclear translocation, baicalein promotes the transcriptional activation of antioxidant response element (ARE)-dependent genes [3]. This leads to the restored expression and activity of critical enzymatic antioxidants, including superoxide dismutase (SOD), catalase (CAT), glutathione peroxidase (GSH-Px), and heme oxygenase-1 (HO-1) [3,4]. Consequently, this re-establishment of redox homeostasis effectively mitigates lipid peroxidation, evidenced by reduced malondialdehyde (MDA) levels, and preserves mitochondrial integrity in PACs [5].

## 6. Challenges in Clinical Translation and Future Perspectives

The extensive preclinical evidence detailed in the preceding sections paints a compelling picture of baicalein as an ideal, multi-target candidate for AP. Despite this promise, advancing these results to human trials necessitates overcoming formidable pharmacological and clinical hurdles.

### 6.1. Preclinical Efficacy and Synergism

The preclinical evidence supporting the therapeutic utility of baicalein in AP is overwhelming in both scope and consistency. This compound consistently demonstrates robust efficacy across diverse etiological models, including those induced by sodium taurocholate, cerulein hyperstimulation, lipopolysaccharide, and hyperlipidemia. Baicalein demonstrates broad-spectrum activity, simultaneously targeting multiple core pathological facet of the disease. As summarized in Table 1, this comprehensive mechanism almost spans the entire pathological cascade. Furthermore, reflecting the “multi-component, multi-target” philosophy of TCM, the efficacy of baicalein is frequently potentiated when administered in combination with other bioactive compounds. A prime example is the combination of emodin and baicalein, which has demonstrated synergistic efficacy in alleviating pathological scores in severe AP rats [39,95]. Significantly, this combination was shown to inhibit the formation of cytoplasmic vacuoles in acinar cells [40]. Beyond this documented pairing, it is plausible to hypothesize that baicalein may exhibit synergy with standard clinical treatments based on mechanistic complementarity. For instance, combining baicalein with secretion inhibitors or protease inhibitors could theoretically provide “upstream-downstream” protection by simultaneously targeting early enzyme activation and the subsequent inflammatory cascade. Consequently, baicalein acts as a versatile foundational agent, effective both as a monotherapy and as a promising component for future multi-target synergistic regimens.

### 6.2. Current Clinical Development

Baicalein is currently positioned at a pivotal juncture in pharmaceutical development as it transitions from foundational safety evaluations to exploratory efficacy trials. Phase I clinical trials conducted in healthy populations have successfully established the safety profile and pharmacokinetics of the oral formulation, specifically baicalein tablets. Randomized, double-blind, single-ascending-dose (SAD) studies have demonstrated that the drug is well-tolerated in humans at doses ranging from 100 to 2800 mg with no serious adverse events reported [97]. Subsequent multiple-ascending-dose (MAD) studies further validate its safety for repeated administration of 200 to 800 mg per day, revealing that the compound does not exhibit significant accumulation in plasma [98,99]. Regarding therapeutic indications, the most advanced clinical progress involves the treatment of respiratory infections, exemplified by a registered multi-center phase IIa clinical trial (NCT03830684) evaluating the efficacy of baicalein in alleviating fever and other symptoms in adults with influenza (Table 2).

However, a significant translational gap persists in the field of AP. Despite overwhelming preclinical evidence elucidating that baicalein targets core lethal mechanisms, including pathological calcium overload and systemic histone toxicity, no specific randomized controlled trials investigating the baicalein monomer for AP patients have been registered in public databases to date. Consequently, as the safety window for high-dose clinical application has been secured by Phase I data, the rigorous clinical validation of baicalein as a targeted therapy for AP remains an urgent and unfulfilled research priority.

### 6.3. Future Perspectives

From a mechanistic standpoint, current evidence remains largely phenomenological, focusing on downstream signaling rather than upstream direct engagement. While specific targets like HMGB1, histones and pancreatic lipase have been structurally validated, a comprehensive map of baicalein’s direct protein interactome is lacking. Future research must prioritize advanced biophysical assays and chemical proteomics to elucidate these precise binding kinetics. Beyond these mechanistic gaps, a distinct translational paradox exists for baicalein: despite robust preclinical efficacy targeting core lethal mechanisms of AP and a well-established phase I safety profile (up to 2800 mg/day), clinical application remains stagnant. Bridging this gap requires addressing three critical challenges. First and foremost, as a classical flavonoid aglycone, baicalein exhibits inherently low solubility in aqueous systems. This fundamental physicochemical limitation significantly restricts its dissolution rate and subsequent bioaccessibility in the human body. When coupled with the compound’s extensive first-pass glucuronidation, these factors collectively limit the systemic bioavailability of the active aglycone. Future development must prioritize advanced delivery systems, such as self-microemulsifying formulations or targeted liposomes, to ensure therapeutic concentrations within the pancreas and gut lymph. Second, a practical disconnect exists between the validated administration route and the pathophysiological reality of AP. While Phase I trials confirmed the safety of oral tablets, patients with severe AP frequently suffer from paralytic ileus and mucosal barrier failure, rendering oral administration unreliable. Furthermore, most preclinical successes rely on prophylactic dosing to block initiating events, whereas clinical patients present with established inflammation. Consequently, translational success hinges on developing stable intravenous formulations and validating efficacy in “delayed-treatment” models, specifically demonstrating the drug’s capacity to reverse established injury rather than merely preventing disease onset. Finally, leveraging its demonstrated synergy with other bioactive compounds, baicalein may yield optimal clinical outcomes when designed as an “add-on” therapy to standard-of-care regimens. Given the established safety window, the initiation of phase II randomized controlled trials is now ethically and scientifically justified to convert this promising candidate into a clinical reality for AP patients.

Extending the therapeutic horizon beyond acute pathology, the therapeutic potential of baicalein in chronic pancreatitis (CP) and its long-term complications warrants further investigation. Research has demonstrated that baicalein effectively inhibits acinar-to-ductal metaplasia (ADM), a pivotal pathological transition in CP that predisposes individuals to pancreatic ductal adenocarcinoma (PDAC) [100,101]. Mechanistically, baicalein suppresses NF-κB activation in acinar cells, thereby preventing their phenotypic transformation from amylase-positive acinar cells into cytokeratin 19-positive ductal-like cells [100]. Additionally, baicalein optimizes the inflammatory microenvironment by curbing the secretion of TNF-α and nitric oxide (NO) from activated macrophages [100]. Although it does not directly modulate the Notch/Hes pathway, its capacity to reshape the inflammatory niche positions baicalein as a promising candidate for halting the progression from chronic inflammation to malignancy [100]. Future clinical research should evaluate whether baicalein intervention can reduce the incidence of ADM and the subsequent risk of PDAC in patients with recurrent or chronic pancreatitis.

## 7. Conclusions

The trajectory of AP from a localized inflammatory event to a systemic catastrophe is driven by a multifaceted interplay of calcium overload, immune dysregulation, and the dissemination of toxic mediators. Baicalein, a pleiotropic flavonoid, distinguishes itself as a unique therapeutic agent capable of targeting virtually every node of this lethal network. Its mechanism of action is profound and comprehensive: it acts upstream to stabilize intracellular calcium homeostasis, centrally to dismantle the inflammatory signaling machinery (TLR4/NF-κB/NLRP3), and systemically to neutralize circulating nuclear toxins (histones) and lipotoxic mediators (lipase/FFAs). This multi-layered protection not only preserves pancreatic acinar integrity but also fortifies distal organs against failure. Currently, baicalein stands at a pivotal translational juncture. While preclinical data are robust and human safety has been validated in Phase I trials, the specific clinical efficacy of baicalein for AP remains untested in randomized controlled settings. The future of baicalein therapy lies in overcoming biopharmaceutical barriers to enhance bioavailability and in designing precision clinical trials that utilize mechanism-specific biomarkers. Given its established safety window and compelling mechanistic rationale, baicalein represents a “ready-for-translation” candidate. Immediate efforts to initiate phase II efficacy trials are scientifically justified and hold the promise of delivering a targeted pharmacological therapy for this debilitating disease.

## Figures and Tables

**Table 1:** Studies of baicalein on treating experimental acute pancreatitis.

Species	Models	Dosage Regimen	Sampling Time	Effects of Treatment	References
SD rats	NaTC (5%, 1 mL/kg, *i.d.*)	20 mg/kg, after model induction, *i.v.*	3, 6, and 12 h after operation	↓ pancreatic TNF-a, IL-6 and MPO ↓ pancreatic SDF-la and SDF-lp	[39]
SD rats	NaTC (5%, 1 mL/kg, *i.d.*)	20 mg/kg, after model induction, *i.v.*	3, 6, and 12 h after operation	↓ mortality at 12 h ↓ pancreatic pathological changes at 6 and 12 h ↓ serum TNF-α and IL-6 at 6 and 12 h ↓ ascites amount at all time points	[96]
SD rats	NaTC (5%, 1 mL/kg, *i.d.*)	3.5, 7, or 14 mg/kg, combined with emodin, after model induction, *i.v.*	12 h after operation	↓ pancreatic histopathology score ↓ serum amylase, TNF-α, and IL-6 ↓ vacuole formation in PACs ↓ NaTC -induced Ca^2+^ overload in PACs	[40]
C57BL/6 mice	CER (5 × 50 μg/kg at 1 h intervals per day for 3 days, *i.p.*)	20 mg/kg per day for 3 days, 1 h after last CER injection, *i.v.*	24 h after the last CER injection on day 3	↓ pancreatic pathological changes ↓ infiltration of macrophages and neutrophils ↓ pancreatic TNF-α, IL-1β, and IL-6 ↓ MAPK, NF-κB, and STAT3 signaling pathways	[38]
SD rats	Fed with high fat diet for 2 w and NaTC (5%, 1 mL/kg, *i.d.*)	20 mg/kg, after model induction, *i.v.*	12 h after operation	↓ pancreatic pathological changes ↓ serum amylase, TG and TC ↓ pancreatic TNF-α, IL-4, and IL-6 ↓ pyroptotic cells	[56]
SD rats	NaTC (3.5%, 1 mL/kg, *i.d.*)	200 mg/kg, after model induction, *i.p.*	12 h after operation	↓ pancreatic and lung pathological changes ↓ serum amylase ↓ TNF-α, IL-1*β*, and IL-6 in pancreas, lung, and BALF ↑ GSH, SOD, and CAT activity ↓ ROS in pancreas and lung ↓ TLR4, MyD88 and TRIF in lung	[35]
PACs (AR42J)	LPS (10 mg/L)	25–75 μM before LPS	24 h after LPS	↑ cell activity ↑ miR-224-5p ↓ IL-1β, IL-6, TNF-α, and IL-18 ↓ PARP1, NF-κB65, p-IκB-α, IL-18R, GSDMD, ASC, NLRP3, and caspase-1 ↓ apoptosis	[37]
C57BL/6J mice	Histones (50 mg/kg, 100 μL, *i.v.*)	20, 200 mg/kg, 30 min before and 1 h after model induction, *i.p.*	4 h after histones infusion	↓ lung pathological changes ↓ serum CK-MB, IL-6 and KC	[41]
C57BL/6J mice	NaTC (3%, 50 μL, 5 μL/min, *i.d.*)	20, 200 mg/kg, 2 h before and 1 h after operation, *i.p.*	24 h after operation	↓ pancreatic pathological changes ↓ serum amylase and lipase ↓ serum levels of cTnT, ALT, AST, urea, and IL-6	[41]
SD rats	NaTC (5%, 1 mL/kg, *i.d.*)	20 mg/kg, *i.v.*	12 h after operation	↓ M1 macrophages in the pancreas ↓ PA-induced macrophage M1 polarization ↓ pancreatic HMGB1, TLR4, and NLRP3 ↓ PACs pyroptosis	[46]

Abbreviations: NaTC, sodium taurocholate; *i.d.*, intra-pancreaticobiliary duct; *i.v.*, intravenous; TNF-α, tumor necrosis factor-α; IL, interleukin; MPO, myeloperoxidase; SDF, stromal derived factor; PACs, pancreatic acinar cells; CER, cerulein; *i.p.*, intraperitoneal; MAPK, mitogen-activated protein kinase; NF-κB, nuclear factor kappa-B; STAT3, signal transducer and activator of transcription 3; TG, triglyceride; TC, total cholesterol; BALF, bronchoalveolar lavage fluid; GSH, glutathione; SOD, superoxide dismutase; CAT, catalase; ROS, reactive oxygen species; TLR4, toll-like receptor-4; MyD88, myeloid differentiation primary response gene 88; TRIF, TIR-domain-containing adapter-inducing interferon-β; LPS, lipopolysaccride; PARP1, ADP-ribose polymerase-1; p-IκB-α, phospho-kappa B alpha; GSDMD, gasdermin D; ASC, apoptosis-associated speck-like protein containing a CARD; NLRP3, NOD-like receptor thermal protein domain-associated protein 3; CK-MB, creatine kinase MB; KC, keratinocyte chemoattractant; cTnT, cardiac isoform of troponin T; ALT, alanine aminotransferase; AST, aspartate aminotransferase; PA, palmitic acid. ↑, increase; ↓, decrease.

**Table 2:** Summary of clinical development and key translational studies of baicalein.

Study Phase	Study Phase	Study Type	Title	Condition
NCT03830684 CTR20182427 ChiCTR1900020928	Phase IIa	Interventional	A Randomized, Double-blind, Placebo-controlled, Multicenter and Phase IIa Clinical Trial for the Effectiveness and Safety of Baicalein Tablets in the Treatment of Improve Other Aspects of Healthy Adult With Influenza Fever	Influenza (with Fever)
N/A (Published [97])	Phase I	Interventional	Safety, tolerability, and pharmacokinetics of a single ascending dose of baicalein chewable tablets in healthy subjects	Healthy Volunteers (Pharmacokinetics/Safety)
N/A (Published [98])	Phase I	Interventional	Multiple-Ascending-Dose Pharmacokinetics and Safety Evaluation of Baicalein Chewable Tablets in Healthy Chinese Volunteers	Healthy Volunteers (Pharmacokinetics/Safety)
CTR20140260 CTR20140261 CTR20140268	Phase I	Interventional	Safety, tolerability, pharmacokinetics, and food effect of baicalein tablets in healthy Chinese subjects: A single-center, randomized, double-blind, placebo-controlled, single-dose phase I study	Healthy Volunteers (Pharmacokinetics/Safety)
CTR20140263 CTR20140267	Phase I	Interventional	Safety, tolerability, and pharmacokinetics of oral baicalein tablets in healthy Chinese subjects: A single-center, randomized, double-blind, placebo-controlled multiple-ascending-dose study	Healthy Volunteers (Pharmacokinetics/Safety)
ChiCTR2000033286	N/A	Interventional	A Randomized, Double-blind, Placebo-controlled trial for determination of the Efficacy and Safety of Baicalein in Hospitalized Adult Mild and Moderate Patients of novel coronavirus pneumonia (COVID-19)	COVID-19

## Data Availability

No new data were created or analyzed in this study.

## Abbreviations

The following abbreviations are used in this manuscript:

AP	acute pancreatitis
PACs	pancreatic acinar cells
ER	endoplasmic reticulum
DAMPs	damage-associated molecular patterns
HMGB1	high mobility group box 1
TNF-α	tumor necrosis factor-alpha
IL	interleukin
SIRS	systemic inflammatory response syndrome
MODS	multiple organ dysfunction syndrome
ALI	acute lung injury
ARDS	acute respiratory distress syndrome
TCM	traditional Chinese medicine
TLR4	Toll-like receptor 4
PAMPs	pathogen-associated molecular pattern molecule
MyD88	myeloid differentiation primary response gene 88
TRIF	TIR-domain-containing adapter-inducing interferon-β
miR	microRNA
NF-κB	nuclear transcription factor-kappa B
MAPK	mitogen-activated protein kinase
ERK	extracellular signal-regulated kinase
JNK	c-Jun N-terminal kinases
JAK2	Janus Kinase 2
STAT3	signal transducer and activator of transcription 3
BALF	bronchoalveolar lavage fluid
ALOX15	arachidonate 15-lipoxygenase
iNOS	inducible nitric oxide synthase
MPO	myeloperoxidase
NETs	neutrophil extracellular traps
ICAM-1	intercellular adhesion molecule-1
FAs	fatty acids
IP3R	inositol 1,4,5-trisphosphate receptor
DARTS	drug affinity responsive target stability
NLRP3	NOD-like receptor family pyrin domain containing 3
PARP1	poly (ADP-ribose) polymerase 1
TXNIP	thioredoxin-interacting protein
Nrf2	nuclear factor erythroid 2-related factor 2
SLC7A11	solute carrier family 7 member 11
GPX4	glutathione peroxidase 4
ACSL4	long-chain-fatty-acid-CoA ligase 4
PUFAs	polyunsaturated fatty acids
MOMP	mitochondrial outer membrane permeabilization
DIABLO	diablo IAP-binding mitochondrial protein
CHOP	C/EBP homologous protein
FFAs	free fatty acids
AIF	apoptosis-inducing factor
ROS	reactive oxygen species
ARE	antioxidant response element
SOD	superoxide dismutase
CAT	catalase
GSH-Px	glutathione peroxidase
HO-1	heme oxygenase-1
MDA	malondialdehyde
SAD	single-ascending-dose
MAD	multiple-ascending-dose
CP	chronic pancreatitis
ADM	acinar-to-ductal metaplasia
PDAC	pancreatic ductal adenocarcinoma
NO	nitric oxide
